# Can policies improve language vitality? The Sámi languages in Sweden and Norway

**DOI:** 10.3389/fpsyg.2023.1059696

**Published:** 2023-03-29

**Authors:** Anika Lloyd-Smith, Fabian Bergmann, Laura Hund, Tanja Kupisch

**Affiliations:** ^1^Department of Linguistics, University of Konstanz, Konstanz, Germany; ^2^Cluster of Excellence “The Politics of Inequality”, University of Konstanz, Konstanz, Germany; ^3^Department of Politics and Public Administration, University of Konstanz, Konstanz, Germany; ^4^Department of Language and Culture, UiT The Arctic University of Norway, Tromsø, Troms, Norway

**Keywords:** language policy, Sweden, Norway, Sámi languages, indigenous languages, revitalisation, language vitality

## Abstract

**Introduction:**

Language policies are often aimed at changing language behaviours, yet it is notoriously difficult to assess their effects. This study investigates language use and competence in the Indigenous Sámi populations of Norway and Sweden in light of the national-level policies the two countries have adopted.

**Methods:**

We provide a cross-country comparison of relevant educational, linguistic and budgetary policies in Sweden and Norway. Next, we present novel data from a survey with 5,416 Sámi and non-Sámi participants in 20 northern municipalities, examining Sámi language use and proficiencies across generations and contexts. Lexical proficiency in North Sámi was tested in a small subset of participants.

**Results:**

Sámi language use has dropped considerably over the past three generations. Only a small proportion of Sámi are highly fluent and use a Sámi language with their children (around 4% in Sweden and 11% in Norway). One fifth of Sámi adults use a Sámi language at least ‘occasionally’, and use is most common in the home context. Sámi language knowledge remains negligible in the majority population.

**Discussion:**

The higher levels of language use and proficiency in Norway seem at least in part to reflect the more favourable policies adopted there. In both countries, more work is needed to increase speaker numbers, also in the majority population.

## 1. Introduction

Attitudes towards multilingualism have changed drastically over the past century. While scholars for many years saw multilingualism as an obstacle, most of the Western world today embraces multilingualism. Linguistic rights have been recognised as human rights, and being multilingual is increasingly viewed as an asset, both for the speakers themselves and for society at large. For example, with the European Charter for Regional or Minority Languages, the Council of Europe (1992) has committed itself to the protection and promotion of regional and minority languages. Likewise, researchers, parents and teachers are nowadays more conscious about the intimate connection between an individual’s mother tongue and identity formation.

In the context of the European Union, Norway and Sweden are both well-known for their high standard of living and commitment to multilingualism, and are relatively advanced in their Indigenous policies (Ingebritsen, 2006; Headey and Muffels, 2021). They are also home to the Sámi languages, all of which are protected by the EU Charter. At the same time, their policy approach has differed. For example, only Norway has ratified the ILO Convention on the Rights of Indigenous Peoples (Convention no. 169), and the Norwegian Sámi Act makes Sámi an official language alongside Norwegian in administrative areas, equal to Norwegian (The Sámi Act, 1987: Ch. 1. section 5). In Sweden, in contrast, the Sámi languages have the status of protected minority languages (Lag, 2009: 724).

Little is known about the effects of these differing approaches on levels of Sámi language maintenance. Further, most work to date has approached the topic from a qualitative or ethnographic perspective (e.g., Johansen, 2013; Sariavaara et al., 2013; Vangsnes, 2022). One reason for the lack of quantitative approaches may be that there are few speakers remaining, making it difficult to carry out large studies (Grenoble and Osipov, 2023). Another point is that, in both Indigenous and in scientific communities, there exists a certain level of scepticism towards measurement and quantification of Indigenous language, resulting from ill-treatment through Westernised measures in the past (e.g., Kahkalau, 2017; McIvor, 2020). While acknowledging the need for sensitivity and cooperation with Indigenous communities, we argue that providing quantitative data on existing language practices is an essential step in formulating recommendations for actions to be taken to facilitate Sámi language learning.

In this paper, we first perform a cross-country comparison of the national-level policies (linguistic, budgetary, and educational) that have been adopted in Sweden and Norway over the past few years. We focus on policies enacted on the national level and their consequences for the formal status of the Sámi languages, as well as for the opportunities to use and learn Sámi in the two countries. Drawing on data from a survey with Sámi and non-Sámi residents in selected northern municipalities, we examine levels of Sámi language use and proficiency. These data were collected in summer 2021 and contain responses from 5,416 respondents in Stage I of data collection, and 918 respondents in Sweden and 502 respondents in Norway in Stage II (see section 3 for details).

We focus on Sweden and Norway (rather than, e.g., Finland and Norway) firstly because they are home to the largest number of North Sámi speakers. Second, from the perspective of politics and political systems, Sweden and Norway share more characteristics with each other, being constitutional monarchies, than with Finland, which is a parliamentary republic (see Wivel and Nedergaard, 2017). Further, the development of the Finnish welfare state was later and followed a different path as compared to the other countries from the so-called Nordic model.^1^

To our knowledge, there exist no large-n cross-country comparisons that investigate the effects of policies on Indigenous language use from a quantitative perspective, nor any investigations on the effects of policies on language maintenance. In this study, language use across the lifespan is measured by means of a questionnaire for adult-aged heritage speakers (Lloyd-Smith et al., 2017; Kupisch et al., 2020),^2^ and objective proficiency is measured by means of a Yes/No vocabulary test (Gyllstad et al., in preparation).^3^ The addition of non-Sámi participants to research in this field is new, and is hoped to provide insights into attitudes and language behaviour in the majority (i.e., non-Sámi) population. The survey also contains data on the topic of Sámi language use across the lifespan, with different conversation partners and, in a smaller subsample, scores from a vocabulary test in North Sámi. We discuss the extent to which the language use and proficiency data in each country may be explained by differences in policies.

## 2. Background

### 2.1. Language revitalization

In work on language revitalization, a number of scales have been developed to assist in operationalising the status of endangered (and non-endangered) languages. One of the most influential scales is Fishman’s Graded Intergenerational Disruption Scale (henceforth GIDS, Fishman, 1991), which classifies the world’s languages on a scale from 1 (vital, national language) to 9 (dormant). The GIDS has remained a central instrument of language policy research, arguably due to its wide applicability across language scenarios, and its effectiveness in highlighting the complex factors that influence language revitalization. In Fishman’s (2004) discussion of the GIDS, he argues that the success of language planning greatly depends on whether or not the measures in question are appropriate for the stage at which the language finds itself. Though the GIDS is not to be understood as a strict hierarchy, Fishman (2004) urges language planners to lend particular importance to Stage 6, which is concerned with intergenerational language transmission and use within the family. If high-level measures—such as those intended to secure greater power or prestige amongst the majority society—are introduced before Stage 6 (and intergenerational mother-tongue transmission is secured), the success of those measures will be short-lived or limited in scope. Fishman gives the example of the Irish language, which received extensive support at the nation-wide level for education programmes, but which today is often first learned as a school language, rather than as a mother tongue (p. 429). At worst, pushing for high-level measures before their time can lead to diminishing trust, and waste of resources that are already in low supply, such as money and time (p. 435). What exacerbates the problem is that, according to Fishman, language planning is difficult *precisely* at Stage 6, since language behaviour at home, with one’s family and with one’s own children, takes place in a realm of ‘spontaneity’ and ‘intimacy’ that is hard to reach with rationally-planned measures (p. 431). Nonetheless, if mother-tongue revitalisation is the goal, then ensuring language use in the family realm is essential.

The GIDS is also useful for our particular case, because it enables a comparison of languages across individual countries. The EGIDS, which is an expanded version of Fishman’s GIDS (Graded Intergenerational Disruption Scale), developed by the Ethnologue, places North Sámi at Stage 2 (‘provincial’) in Norway, meaning that language is used in education, work, mass media and government within administrative subdivisions. The EGIDS is a scale that operationalizes 11 different levels of language engdangerement, with ‘international’ (0) at the top, representing the most vital languages, and 10 at the bottom (extinct). In Sweden, North Sámi is placed much further down the scale at Stage 6 (threatened), meaning that the language is used for face-to-face communication, but that it is losing users’ (Eberhard et al., 2022). The implications of Stage 6 are, as mentioned above, that the focus should be on intergenerational transmission (Eberhard et al., 2022). If we were to draw implications from this classification, it would be that higher-level revitalization measures for North Sámi should be possible in Norway as compared to Sweden.

### 2.2. The Sámi languages and speakers

The exact number of ethnic Sámi is unknown, since information on language and ethnicity is not collected by Sweden or Norway in the national census. Estimates range between 65,000 and 100,000, though only a minority is still able to speak a Sámi language, with estimates ranging between 18,000 and 30,000 speakers, mostly in Norway (Aikio-Puoskari, 2009). While all nine remaining Sámi languages are endangered (UNESCO), North Sámi is the most vital and has the largest estimated number of speakers (~20,000). The Endangered Languages Project (ELP) considers North Sámi vulnerable, and the other Sámi languages endangered to varying degrees (Todal, 2013; Hettema and Outakoski, 2020; Vangsnes, 2022). Geographically, about two thirds of the North Sámi speakers live in northern Norway, and a smaller share lives in northern Sweden (~5,000–7,000; Rasmussen and Nolan, 2011) and Finland (~2,000; Rasmussen and Nolan, 2011). Besides North Sámi, the other Sámi languages spoken in Sweden and Norway include South Sámi, spoken by 500–1,000 people (Hettema and Outakoski, 2020), and Pite and Lule Sámi, which are spoken by only a small handful of individuals (Hettema and Outakoski, 2020). The Sámi languages enjoy the highest levels of vitality in the Sámi administrative areas, which exist in Norway, Sweden, and Finland. These territorially-confined areas consist of municipalities in which extended language rights apply (described in section 2.3.).^4^ The purpose of the administrative areas is to provide resources and opportunities beneficial for the survival and advancement of the Sámi languages. For example, state-run schooling through the medium of Sámi exists in all Sámi administrative areas (Huss and Lindgren, 2022), as does the right to communicate with the authorities in a Sámi language (Svonni, 2008; Aikio-Puoskari, 2009). However, there are no monolingual speakers remaining; Sámi speakers are bilingual with the majority language and a Sámi language (Aikio-Puoskari, 2009).

Today, the Sámi people are largely assimilated and do not differ from the majority in terms of economic measures, unlike most other Indigenous populations world-wide (Yasar et al., in preparation).^5^ Nonetheless, the Sámi consistently report experiencing higher levels of discrimination than the majority population (see Hansen et al., 2008, 2016, for Norway, and Yasar et al., in preparation, see footnote 5, for Sweden). Therefore, despite socio-economic equality, inequalities seem to persist. Language is one area where this becomes apparent: A recent study by Yasar et al. (in preparation, see footnote 5) showed that speaking Sámi outside the family context was related to a higher proportion of perceived discrimination. Such findings may reflect a higher sensitivity to missing opportunities for engagement with Sámi issues. For example, not all Sámi children have access to education through the medium of Sámi (see section 2.3.2). For those who do, it is common to discontinue education in Sámi and to pursue secondary education in the majority language (Vangsnes, 2021). Based on studies on small speaker communities, it would seem that language shift from Sámi to the majority languages is still ongoing. In a study on reindeer-herding populations in Sweden, Svonni (2008) evidenced North Sámi proficiency in 60% of the older generation (over 60 years), dropping to 45% in the 30–59 year-olds, and 20% in the younger generation. However, no quantitative data on today’s parents and their language use patterns with their children exist.

### 2.3. Sámi-directed policies in Sweden and Norway

In the following, we outline the differences in national-level policies across the domains of language, education, and budgetary spending to provide a picture of the overall language policy context. Although the implementation at local or municipality level might vary, we chose to focus on national-level policies because they define the general scope of which language measures can be implemented. Further, our data would not be suited to a municipality-level comparison since we do not have an even distribution of participants across municipalities, and many of the participants no longer live in the areas they grew up in.

#### 2.3.1. The status of Sámi in language

As one measure to improve the vitality of the Sámi languages, both Sweden and Norway endorse instruments of international law. For example, they voted in favour of the United Nations Declaration on the Rights of Indigenous Peoples (UNDRIP, 2007) and ratified the European Charter for Regional or Minority Languages (ECRML)^6^ (Council of Europe, 1992). They require states to provide conditions that protect their Indigenous populations’ languages. Consequently, the Sámi languages enjoy the status of officially recognised languages in both countries. However, their status vis-à-vis the majority languages, i.e., Norwegian and Swedish, and the specific conditions for their protection differ between Norway and Sweden.

In Sweden, Sámi is considered a national minority language alongside Finnish, Yiddish, Meänkieli, and Romani (see Lag, 2009: 724, on national minorities and minority languages). The state has the responsibility to protect and promote these languages. At the same time, Swedish legislature explicitly names Swedish as the main language (*huvudspråk;* see Lag, 2009, p. 600), thereby establishing a hierarchy between Swedish as the main language and protected minority languages (including Sámi). By contrast, the Act relating to Language (the Language Act) (2022) gives a higher status to Sámi as compared to other minorities, making the state responsible for ‘using, developing, and strengthening’ both the Norwegian (Bokmål and Nynorsk) and the Sámi languages, while for the other protected minority languages (Kven, Romani, and Norwegian Sign Language) this responsibility is narrowed to ‘protecting’ and ‘promoting.’ Even though the Norwegian Language Act states that ‘Norwegian is the primary national language in Norway’ Act relating to Language (the Language Act) (2022), the Sámi languages as ‘Indigenous languages’ Act relating to Language (the Language Act) (2022) enjoy a special status. In addition, the The Sámi Act (1987) makes Sámi an official language in the administrative areas, alongside Norwegian, and states that ‘Sami and Norwegian are languages of equal worth.’ Hence, while Norwegian Sámi language policies are based on the principle of Sámi and Norwegian having an equal standing, the Swedish approach features a more hierarchical distinction between Sámi as one of several minority languages and Swedish as the paramount language.

These differences are also apparent when considering the policies applied in the administrative areas for the Sámi languages. In Sweden, Sámi speakers living in the respective area have the right to use Sámi when communicating with public authorities. These authorities, in turn, are obliged to reply in Sámi orally. Unfortunately, despite their right to use Sámi, the speakers might end up using their minority language for fear of misunderstandings or shame (Elenius and Ekenberg, 2002). In Norway, policies provide conditions exceeding those in Sweden (see The Sámi Act, 1987, Chapter 3). Here, in the administrative area, Sámi speakers have the right also to receive written answers in Sámi by public authorities, which are in addition obliged to provide any laws, regulations, announcements, or forms that are of relevance for the Sámi both in Norwegian and Sámi. Moreover, there is a right to receive services in Sámi by any public social and health institution and the Church of Norway. Sámi has virtually the same official status as Norwegian in the public domain within the administrative area. Within the Swedish equivalent, the terms on which Sámi can be used are more narrowly defined.

#### 2.3.2. Sámi education policies and schooling

With regards to Sámi education policy, Norway first introduced (North) Sámi language classes at schools in the 1950s, mainly in order to transition pupils to the Norwegian curriculum (see Sollid, 2022, for an overview of the Norwegian Sámi curricula). A small number of schools taught South Sámi; Lule Sámi was introduced later, in the 1990s (Todal, 1998). A curriculum for Sámi as a subject was introduced in 1974, and a parallel Sámi curriculum just ‘3 years’ later. Today, school students in Norway can choose between three streams of Sámi learning: Sámi as a first language, as a second language, or as a foreign language (Vangsnes, 2022). The right to Sámi as a medium of instruction extends until Year 10 (Education Act, 1998, Ch. 6, Sec. 6–3). Further, the *Sámi University of Applied Sciences* in Kautokeino, founded by the Norwegian Ministry of Education in 1989, became the first and only tertiary education institution with Sámi as the main language, and also provides Sámi training to teachers from all four countries. According to Vangsnes (2022), there were a total of 2,336 pupils learning North, Lule and South Sámi in Norway in 2009 (across all three streams), while in 2021 that number had risen minimally to 2,522.

In Sweden, the right to mother tongue instruction, established in 1976, stipulates that every pupil has a right to receive language tuition in their native language. The teaching of Sámi as a school subject began in Sweden in 1977, and a full school curriculum was introduced in 2011. As with Norway, Sámi can be learned in three different streams. However, Sámi as a medium of instruction is available at a total of five schools in Swedish Sápmi, and only until Year 6 (Belančić and Lindgren, 2020). According to a recent report by Hettema and Outakoski (2020), a total of 978 school-aged pupils were learning Sámi in Sweden in 2020, and close to 200 further pupils were receiving distance education from various providers. Distance learning plays an important role in increasing numbers of pupils learning Sámi in Sweden.

To provide some means of comparison, and since most of our participants were from Troms og Finnmark (Norway) and the Norbotten County (Sweden), we compiled an overview of preschool and lower secondary school pupils that had Sámi as their language of tuition in 2020 (Table 1). In brackets are the total number of children enrolled in the respective school level. As shown by our calculations, proportionally, there are about three times as many pupils receiving tuition in Sámi at the primary and lower secondary school level in Troms og Finnmark than in Norrbotten. At the preschool/kindergarten level, there are five times as many children receiving Sámi education in Troms og Finnmark. Even when taking into account that there are more Sámi in Norway than in Sweden, the differences are unambiguous.

**Table 1:** Children with Sámi as language of tuition in 2020.

	Troms og finnmark (Norway)	Norrbotten (Sweden)
Preschool/kindergarten	664 (5.87%)	122 (1.13%)
Primary and lower secondary school	596 (3.23%)	200 (1.09%)

Primary and lower secondary education covers 7 years of schooling in both Norway and Sweden. However, the grades are divided into year 1 to 7 (Norway) and 0 to 6 (Sweden), respectively. Sources: Sameskolstyrelsen, Skolverket (2020), Statistics Norway, Statistics Sweden, own calculations.

In summary, Norwegian policies assign the Sámi education plan a status equal to that of the Norwegian mainstream curriculum. In Swedish curricula, Sámi education matters are characterised as supplementary rather than equal to the mainstream curriculum. Historically, the Norwegian Sámi education policies were implemented earlier than in Sweden, with three decades lying between the introduction of a Sámi-medium curriculum in 1977 in Norway and in 2011 in Sweden. A further difference is that Norwegian policy foresees Sámi-medium instruction until grade 10, so for 3 years longer than in Sweden. A challenge for both countries remains finding sufficient teaching staff for the Sámi languages, in particular outside the administrative areas (Anaya, 2011; Hettema and Outakoski, 2020). In both countries, student numbers enrolled in Sámi learning are quite low, but higher in Norway than in Sweden.

#### 2.3.3. Financial resource allocation for the Sámi languages

The different levels of political esteem for the Indigenous languages can also be observed more tangibly by looking at the financial resources allocated to protect and promote the Sámi languages.

In both countries, the Sámi Parliaments play an important role in promoting the languages. The Sami Parliaments, publicly elected by the Sámi, deal with different matters concerning the Sámi people, including language. For example, it is stated in the Swedish Sámi Parliament Act (Sametingslag, 1992: 1433) that the Sámi Parliament shall establish objectives for and lead the Sámi language work, as well as contribute to the development of the use of the Sámi languages. Although the measures for the promotion and protection of the languages are not exclusively funded by the Sámi Parliaments, a comparison of the amounts and the share of the total budget that the respective Sámi Parliament specifically earmarked over the past 5 years for this purpose gives a rough idea of the financial support the Sámi languages receive in both countries. As shown in Table 2, the total budget for the Norwegian Sámi Parliament has increased steadily over the last 5 years, amounting to 54.6 million euros in 2022. The share of the budget specifically earmarked for the categories ‘language’ and ‘culture’ including, for example, expenses for the facilitation of the use of Sámi languages, the integration of the Sámi languages in official information or expenses for Sámi libraries or literature (Sametingets budsjett, 2022), has also increased steadily in recent years, reaching almost 50% (27.1 million euros) in 2022.

**Table 2:** Budget for Sami Parliament Budget in Norway and Sweden from 2018 to 2022 (EUR thousand).

Country	Year	Total budget	Budget for language and culture	Percentage of expenses for language and culture
Norway	2018	45,966	20,945	45.57%
	2019	47,662	21,891	45.93%
	2020	49,737	23,254	46.75%
	2021	53,073	24,439	46.05%
	2022	54,637	27,108	49.61%
Sweden	2018	19,651	4,465	22.72%
	2019	19,998	4,608	23.04%
	2020	34,514	8,665	25.11%
	2021	41,435	8,796	21.23%
	2022	48,093	8,947	18.60%

Sources: Sámediggi (2017) Sametinget budsjett 2018; Sámediggi (2018) Sametinget budsjett 2019; Sámediggi (2020) Sametinget reviderte budsjett 2020; Sámediggi (2020) Sametinget budsjett 2021; Sámediggi (2021) Sametinget budsjett 2021; Sámediggi (2019a) Budgetunderlag 2020–2022.

The total budget available to the Swedish Sámi Parliament is smaller. While it has more than doubled in the last 5 years reaching approximately 48 million euros in 2022, the proportion dedicated to the budget areas ‘culture’ and ‘measures for national minorities’ has decreased from 22.7% in 2018 to 18.06% (8.9 million euros) in 2022 (Sámediggi, 2019a). These budget areas include language expenditures, but not as an individual budget item but as one of many aspects of minority promotion. Overall, expenses for culture and minority promotion in general seem to play a minor role, as only about one fifth of the total budget is earmarked for them, while about half of the total budget is allocated to the promotion of reindeer husbandry. However, the Swedish Sámi Parliament does not define these budgetary allocations. In contrast to its Norwegian counterpart, most of the funds the Swedish Sámi Parliament receives from the government are already pre-allocated and limited in how they can be used (Josefsen et al., 2015). The fact that there is no single appropriation for language is seen by the Sámi Parliament itself as a perturbing hurdle for its linguistic endeavours (Sámediggi, 2019b).

#### 2.3.4. Research questions

The language, educational and budgetary policies outlined above show that both countries are committed to supporting the Sámi languages. At the same time, indicators from the areas of language status planning, educational policy and budgetary spending suggest that the situation in Norway is more favourable. We are interested in the extent to which such conditions are associated with differing levels in Sámi language vitality and use. The research questions for the paper are the following:

**RQ1:**To which extent has Sámi language use changed across the generations?

**RQ2:** What are the self-assessed proficiencies of non-Sámi respondents?

**RQ3:**What are the proficiencies (self-assessed and productive) of ethnic Sámi respondents? How frequently is Sámi used across the lifespan, and in which contexts?

**RQ4:**What differences do we find between Sweden and Norway? To which extent can they be explained by the respective policies adopted at the national level?

## 3. Methods

### 3.1. Ethics approval and procedure

While designing the survey, we actively sought feedback from local stakeholders (such as government representatives) and scholars working in the field. Participation was voluntary and participants could withdraw from the survey at any time. All data was fully anonymized, and will be made freely available to the community in the near future.

Data collection took place in two stages in 15 northern municipalities in Norway and 5 in Sweden in summer 2021. Stage I of data collection consisted of short, computer-assisted telephone interviews; Stage II was an online survey, described in more detail below. The 20 municipalities were selected based on the relatively high share of residents on the electoral role for the Sámi Parliament elections in 2017, which was substantially higher in these municipalities than in others. In the 15 Norwegian municipalities we selected, the mean share of residents on the Sámi electoral role was 20.9% (min = 8.5% in Alta, max = 68.6% in Kautokeino). In the five Swedish municipalities the mean was 9.1% of residents (min = 7.5% in Kiruna, max = 15.5% in Jokkmokk).

### 3.2. Stage I of data collection (CATI)

Stage I of data collection consisted of a short, representative survey carried out by local survey companies in the form of computer-assisted telephone interviews (CATI), and contained questions on demographics, ethnic self-identification, and language use. The companies employed trained interviewers who conducted the interviews in Norwegian and Swedish, respectively. Participants were offered the opportunity to schedule an interview in North Sámi, though no one chose this offer. No sampling of respondents took place. Instead, Stage I was a full population survey in which the companies tried to reach out to every adult with a phone number registered in the selected municipalities. In Norway, our local cooperation partner had access to 17,096 eligible adults with a telephone number registered in the selected municipalities. They managed to reach 11,153 people, of whom 21.5% (*N* = 2,396) agreed to participate in the CATI. The Swedish survey company had access to 22,073 people. They managed to reach 6,265 people on the phone, of whom 48.2% (*N* = 3,020) participated in Stage I.^7^ As shown in Table 3, in Norway, a total of 2,396 telephone interviews were carried out, of which 1,072 (44.7%) were conducted with ethnic Sámi respondents. In Sweden, 3,020 people participated, 847 of whom (28.1%) were ethnic Sámi. Though there are many approaches to defining ethnicity, we defined having a Sámi ethnicity as fulfilling one or more of the following criteria: (i) self-identification as Sámi, (ii) one parent or grandparent with Sámi ethnicity, and/or (iii) a Sámi language was spoken at home. Others were defined as having non-Sámi ethnicity.

**Table 3:** Participation rates by survey stage, country, and variables of interest.

	Norway *N* (of those ethnic Sámi)	Sweden *N* (of those ethnic Sámi)	Variables of interest
STAGE I CATI	2,396 (1,072)	3020 (847)	- Sámi language use (grandparents, parents, and participants themselves)
STAGE II SURVEY	502 (239)	918 (263)	- Demographics (age, gender, municipality) - Self-assessed Sámi language proficiency - Schooling in Sámi
**Ethnic Sámi participants only:**			
	239	263	- Language use across the lifespan - Within the family, by family member - Outside the family context
STAGE II SURVEY	65	82	- Proficiency measure (North Sámi Vocabulary task; NSVT)

**Figure 1:**
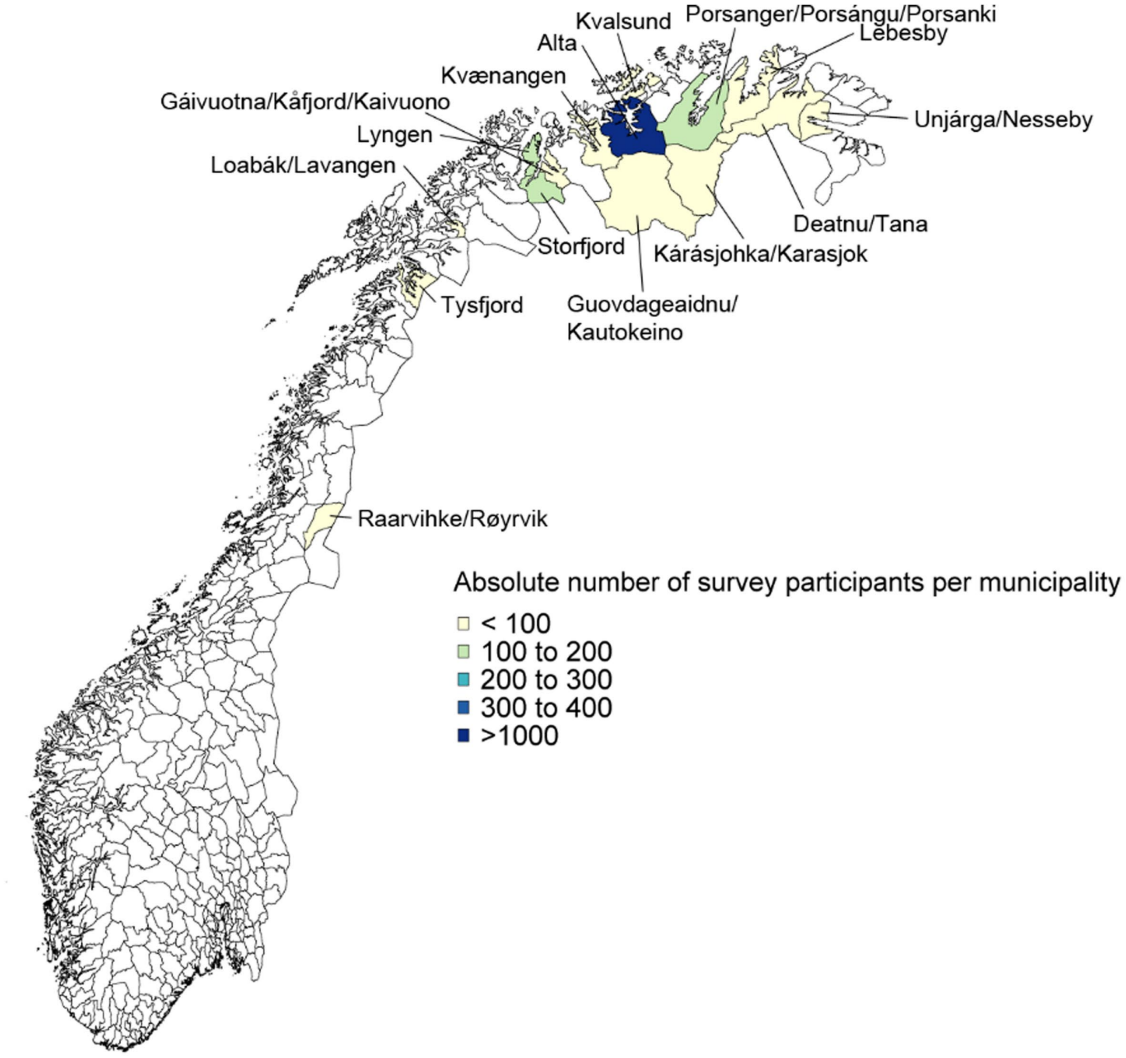
Respondent numbers in Norway.

**Figure 2:**
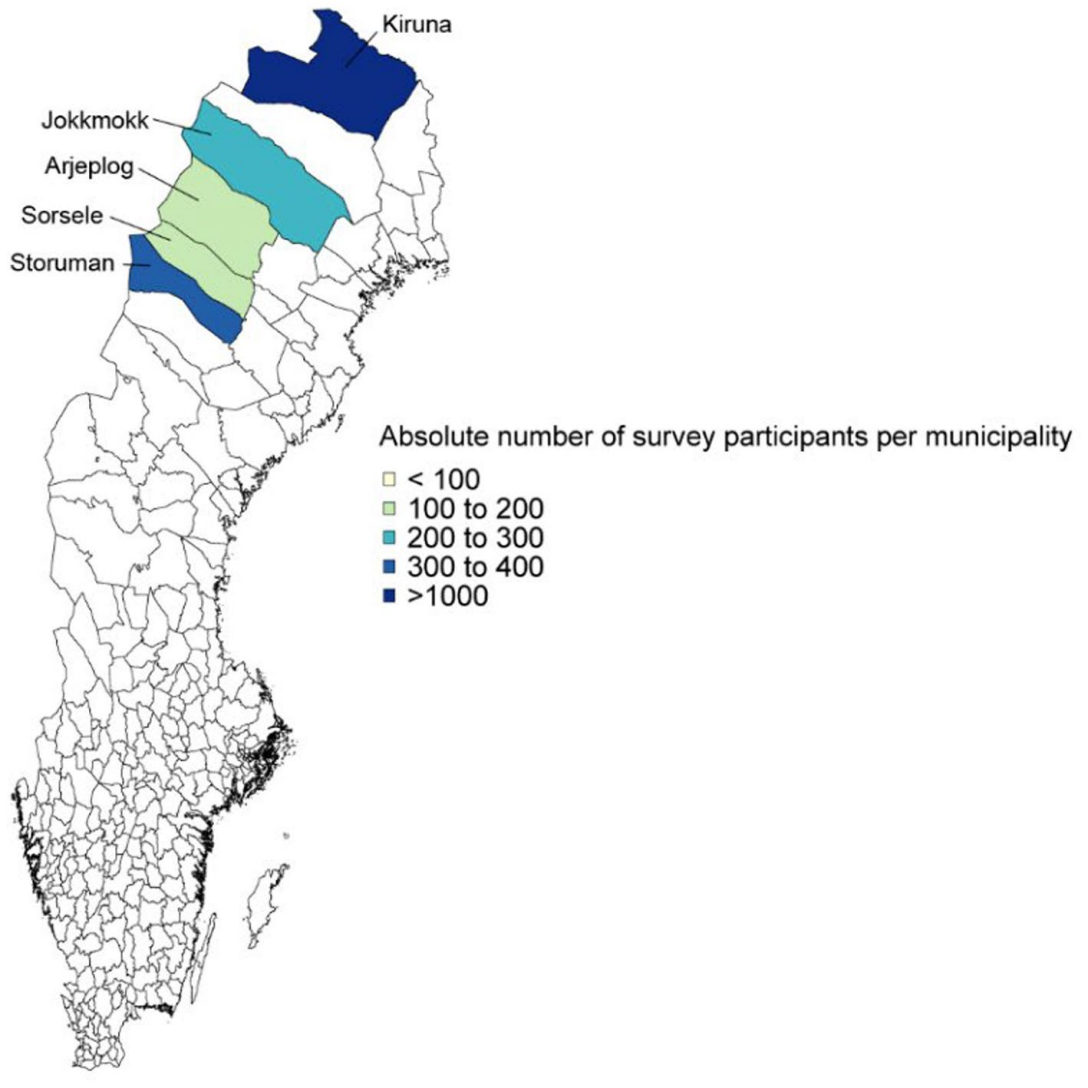
Respondent numbers in Sweden.

The maps in Figures 1, 2 display the number of responses from the 20 municipalities in Norway and Sweden. The vast majority of respondents came from Alta (Norway) and Kiruna (Sweden), as indicated by the shaded areas in dark blue. These large shares reflect the fact that these two municipalities have the largest populations; in fact, the relative share of the population that participated in the survey is reasonably evenly-distributed across the municipalities (see Supplementary materials for the participation shares).

### 3.3. Stage II of data collection

Stage II of data collection consisted of an online survey^8^ with participants who were recruited during Stage I. The questionnaire was available in three languages: the majority language (Swedish or Norwegian), North Sámi, and in English. A total of *n* = 502 (including 239 ethnic Sámi) participated in Stage II in Norway, and *n* = 918 (including 263 ethnic Sámi) in Sweden. All participants regardless of ethnicity answered questions on demographics, self-assessed Sámi proficiency, and Sámi language learning at school. Ethnic Sámi respondents (identified as such in Stage I) received a longer version of the questionnaire that contained questions about Sámi language use across the lifespan and ended with two languages tasks, including a vocabulary test and a speech recording. Participants received a voucher for their participation, and a second one for completing the language tasks.

## 4. Results

In this section, we report on results from the CATI telephone interviews (4.1.), followed by the data from the Stage II survey (section 4.2.) and the North Sámi vocabulary test (section 4.3.). Each section deals with one of the four RQs. The significance of between-country comparisons is explored by means of Chi squared tests of independence (the full data and results for which can be found in Supplementary materials).

### 4.1. Data from telephone survey (CATI)

To examine how Sámi language use has changed across the generations (RQ1), we draw on data from the Stage I CATI interviews. In the telephone interviews, participants were asked whether they themselves, their parents, or grandparents used a Sámi language at home. As shown in Table 4, the proportion of respondents’ grandparents who used Sámi is high, but higher in Norway (75.60%) than in Sweden (56.54%). A Chi-squared test showed this difference was highly significant (χ^2^ = 75.1307, *p* < .001). The number of respondents who report using Sámi today is much lower, at 21.80% in Norway and 20.60% in Sweden (this difference was not significant; χ^2^ = 0.4051). These results suggest that the large gap that was apparent between the two countries for Sámi use amongst grandparents has disappeared for Sámi speakers today. This cannot be due to the age of the respondents, which was similar in Norway (mean = 53.16, sd = 14.37) and Sweden (mean = 52.13, sd = 16.31).

**Table 4:** Use of the Sámi language at home, ethnic Sámi participants (Stage I, CATI).

	Sweden	Norway
Grandparents used Sámi language at home	458 (56.54%)	787 (75.60%)
Parents used Sámi language at home	260 (31.21%)	473 (44.66%)
Participant uses/used Sámi at home	173 (20.60%)	233 (21.80%)

A potential explanation might be that many of the respondents are from Alta (Norway) and Kiruna (Sweden). These are relatively urban areas, where mixed-ethnicity households are probably more prevalent, making the use of Sámi at home less likely (*cf.* Trosterud, 2008). Further, there has been considerable migration to Alta from rural parts of the so-called ‘core area’ in Norway (Melhus et al., 2020). These areas have been less affected by the assimilation and modernization policies (e.g., Trosterud, 2008; Rasmussen and Nolan, 2011; Hermansen and Olsen, 2020). Hence, if the Norwegian parents or grandparents were from areas such as Tana, Karasjok or Kautokeino, they would have been more likely to use a Sámi language. In Sweden, by contrast, language shift seems to have been more sweeping, and even grandparents in the rural inland might have been less likely to use Sámi at home. Further, this data does not show how *frequently* Sámi is used by the individual. More refined measures of language use from the Stage II survey are reported in what follows.

### 4.2. Stage II survey data

#### 4.2.1. Self-assessed proficiency

**Figure 3:**
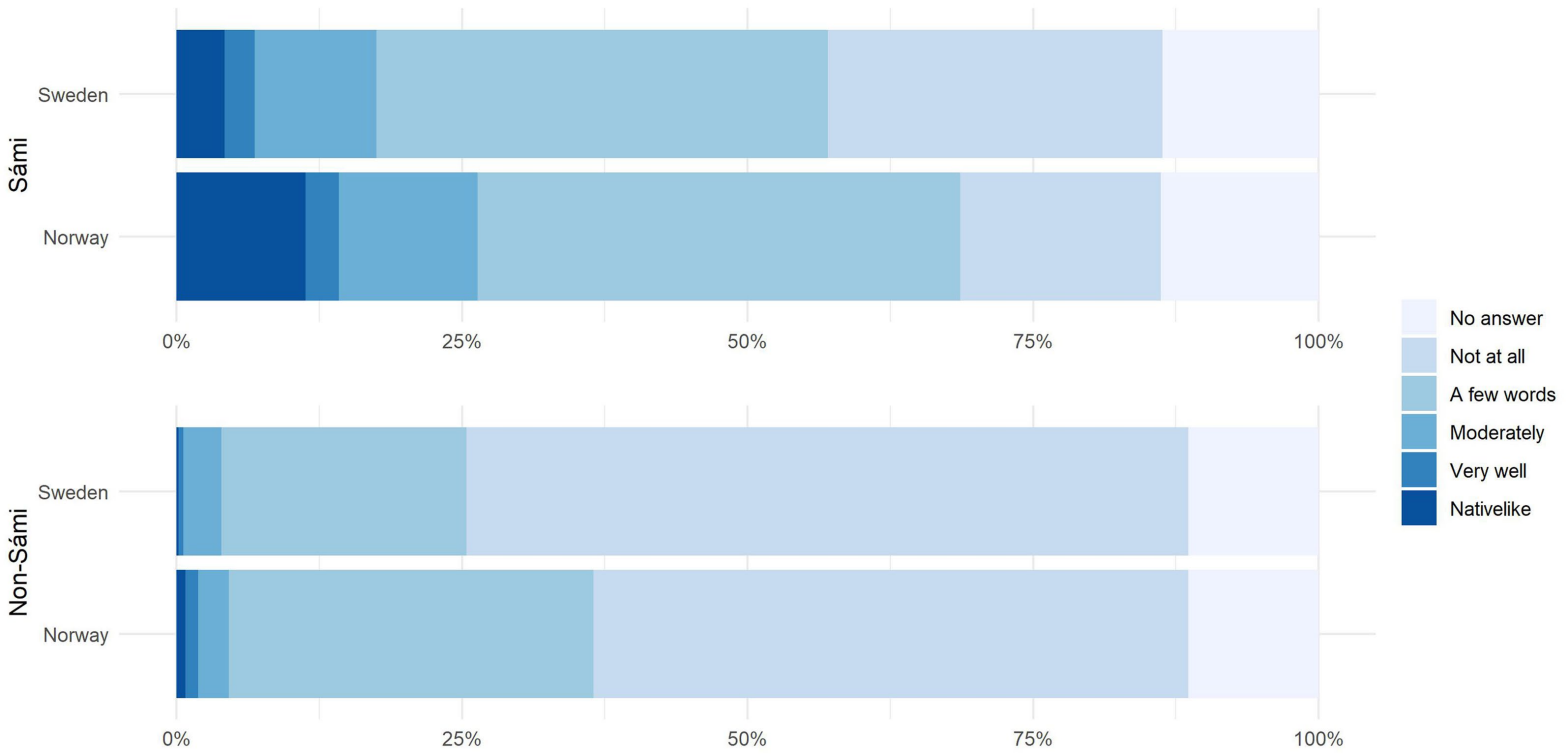
Responses by country and ethnicity to question ‘How well do you understand a conversation in Sámi?’

RQ2 was concerned with the levels of self-assessed proficiency in non-Sámi respondents. We asked all Stage II participants, i.e., Sámi and non-Sámi, to self-assess their proficiency on a scale from 1 = ‘not at all’ to 5 = ‘native-like.’^9^ Figure 3 shows the responses to the question ‘How well can you understand a conversation in Sámi,’ with the answers from the non-Sámi respondents displayed in the top bar, and the Sámi respondents in the bottom bar. In Sweden, a total of 24% of respondents with no Sámi background said they could understand ‘a few words’ or better. In Norway, 36% of respondents said they could understand a few words. A chi-squared test showed that the difference between the two countries was highly significant (χ^2^ = 16.7006, *p* < .01).

Amongst the ethnic Sámi group, 57% in Sweden and 69.25% in Norway said they could understand at least a few words. A moderate to native-like understanding (i.e., a comprehension good enough to follow a conversation) was reported by 18% (Sweden) and 26% (Norway). Once again, the between-country comparison was highly significant (χ^2^ = 16.1118, *p* < .01).

#### 4.2.2. Sámi in schools

**Figure 4:**
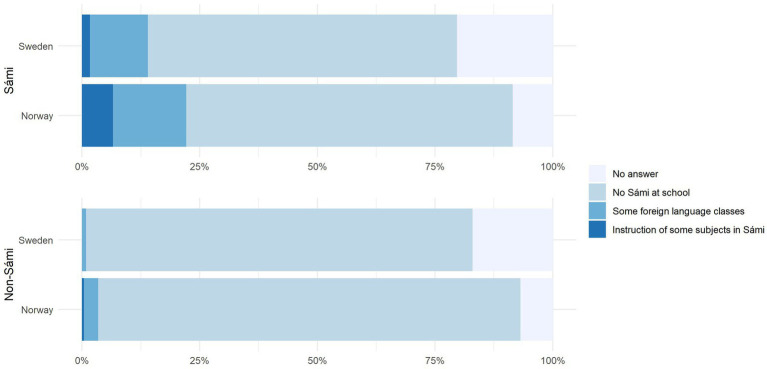
Self-reported Sámi learning at school by country and ethnicity.

Though not directly related to our RQs, we asked participants if they learned Sámi at school, either as a taught language or as a medium of instruction (Figure 4). In Sweden, 12.29% of ethnic Sámi reported learning Sámi, but only 1.69% had Sámi as a medium of instruction. In Norway, 15.75% of ethnic Sámi reported learning Sámi as a foreign language, and 6.6% of received instruction in a Sámi language. The proportion of the majority population who reported having learned Sámi at school was negligible in Sweden (<1%) but noticeably higher in Norway (3.43%). The differences between the two countries become most apparent at the level of Sámi medium education, the numbers for which are almost four times higher in Norway. A chi-squared test showed that this comparison was highly significant (χ^2^ = 18.4291, *p* < .001). The comparison between non-Sámi respondents reached significance also, though this has to be treated with caution due to the low number of data points (Supplementary materials).

A small handful of respondents additionally reported whether they had learned Sámi at primary and/or at high school. In Sweden, only 7 of the 27 respondents continued with Sámi classes (of any kind) at high school level, in Norway, 19 out of 42 respondents did so. It is difficult to know whether these numbers reflect current drop-out rates, since the average respondent went to school in the 1970s and 1980s. However, a recent study showed that numbers of enrolments in North Sámi classes at school in Norway drop by between 30% and 40% between primary and high school (Vangsnes, 2021). These numbers show that making Sámi classes at high school level attractive for students, who are under pressure to perform well in other subjects, remains a challenge.

#### 4.2.3. Sámi language use at home and outside the family

In the following, we examine how frequently ethnic Sámi participants use a Sámi language at home, in other contexts, and across the lifespan (RQ3). We will also examine differences between Sweden and Norway (RQ4). See Table 5 for an overview of participants and their demographics.

**Table 5:** Overview of ethnic Sámi participants in Stage II survey.

	Sweden (*n* = 263)	Norway (*n* = 239)
Gender m/f/other	145/117/1	135/104/0
Mean age/min/max	51.47/19/82	52.7/19/83
**Age of onset in Sámi**		
Ages 0–4 (early bilinguals)	64	80
>Age 5 (late bilinguals)	69	47
No answer	133	112

*Note:* This group mostly included participants with an age of onset over age 10.

**Figure 5:**
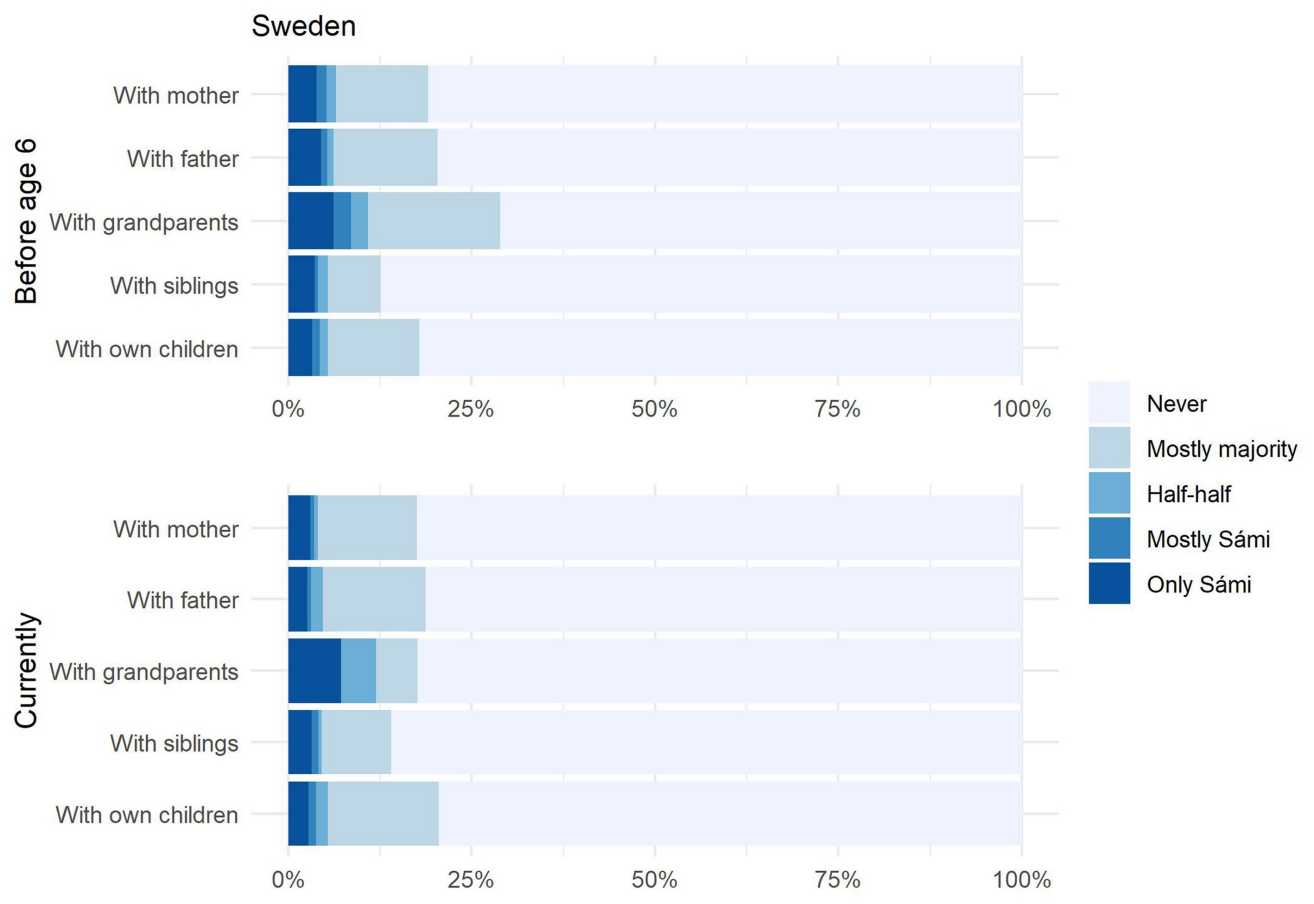
Sweden: Amount of Sámi spoken by family member before starting school vs. currently.

**Figure 6:**
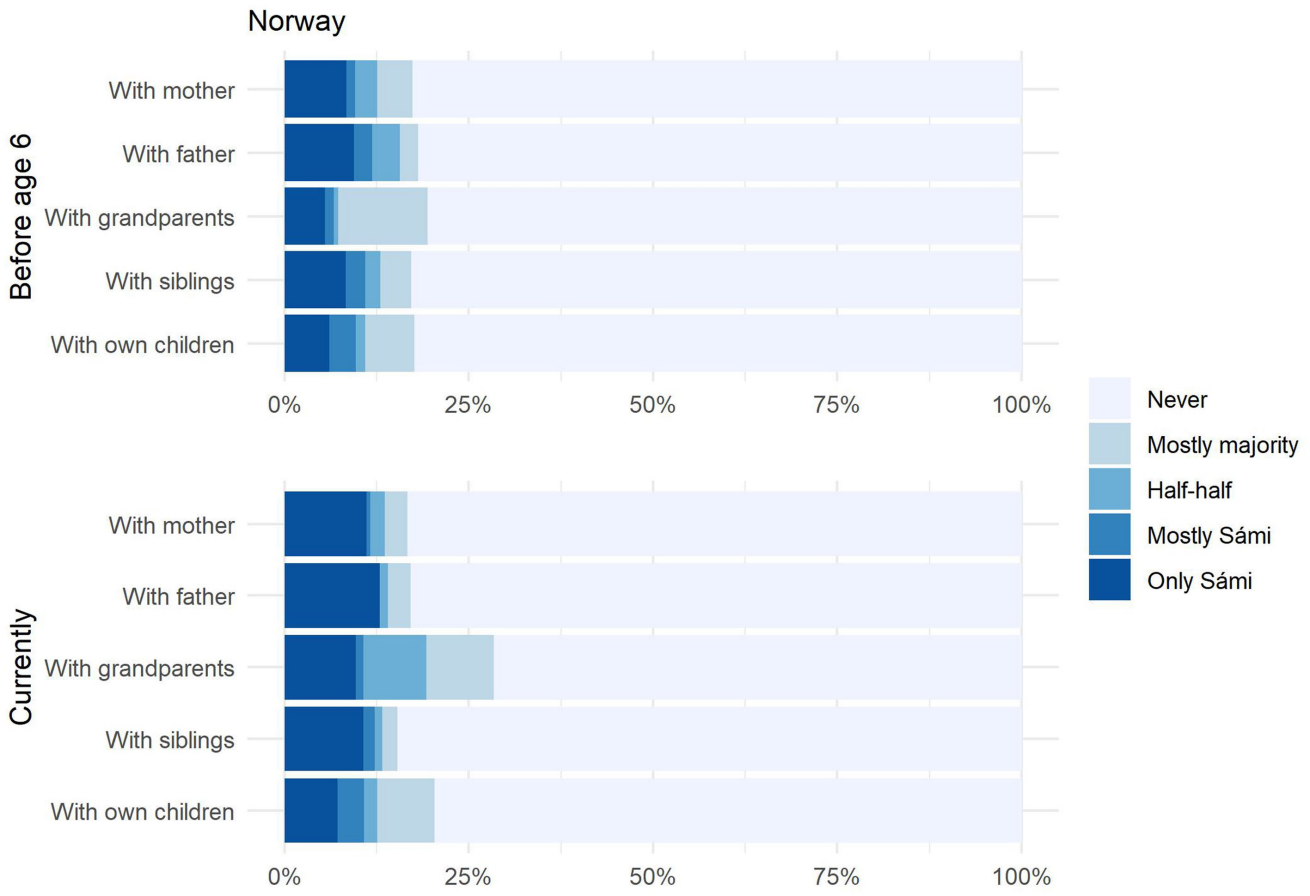
Norway: Amount of Sámi spoken by family member before starting school vs. currently.

First, we turn to language use patterns within the family, the domain identified by Fishman (2004) as central to revitalisation. We asked participants how frequently respondents used a Sámi language with each family member (mother, father, grandparents, siblings, and own children, if applicable) in different periods of their lives (‘before age 6,’ ‘after age 6,’ and ‘currently’). As shown in Figure 5 (Sweden) and Figure 6 (Norway), the overall proportion of Sámi respondents who use Sámi at least occasionally (i.e., they responded ‘mostly majority’) lies at around 20% for both countries. In the same vein, the number of Sámi respondents who ‘never’ use Sámi (indicated by the grey bars) is similar for both countries and lies at around 80%. It is also interesting to note that ‘use’ does not seem to differ across family members, except for a minimal increase for grandparents.

Let us zoom in on those 20% of respondents who answered they use ‘mostly majority’ or better. We will refer to these respondents as ‘Sámi users,’ since even those who responded ‘mostly majority’ can be assumed to use a Sámi language in some form, even if this is restricted to occasional code-switches into a Sámi language while speaking Swedish or Norwegian. As shown in Figure 5, Swedish Sámi users tend to use Sámi only ‘occasionally,’ shown by the yellow bars. In contrast, over half of Norwegian Sámi users (equivalent to around 10% of all Sámi respondents) indicated that they used ‘mostly’ or ‘only’ Sámi with these family members, shown by the blue bars. These data show that there is similar proportion of ‘Sámi users’ in both countries, but that fluency levels are higher amongst Sámi users in Norway. We calculated cross-country comparisons for each family member separately, again using Chi-squared tests. Almost all comparisons were significant (Supplementary materials), signalling a statistically higher amount of use in Norwegian Sámi families than in Swedish Sámi families. Interestingly, one comparison did not reach significance: namely the amount of Sámi used with one’s own children before age 6. We would interpret this to mean that fluent speakers of Sámi in *both* countries are highly interested in passing the language on to their children. This is shown by the observation that a similar proportion of parents in both countries speak ‘only Sámi’ with their children before age 6. However, it may be the case that the conditions in Norway are simply better for doing so, which may explain the gap that appears between the two countries in the number of parents *still* using Sámi with their children in later years of life. In other words, parents in Sweden may more frequently be forced to give up speaking a Sámi language with their children than is the case in Norway, due to a lack of family-based, societal and/or institutional support.

We can also view the respondents’ own language use longitudinally by comparing language use with their own mother and father before age 6, with language use with their own children currently. Here, we see only a minimal decrease, suggesting that language use in today’s Sámi speakers remains stable across the lifespan. This seems to constitute a shift in a positive direction.

**Figure 7:**
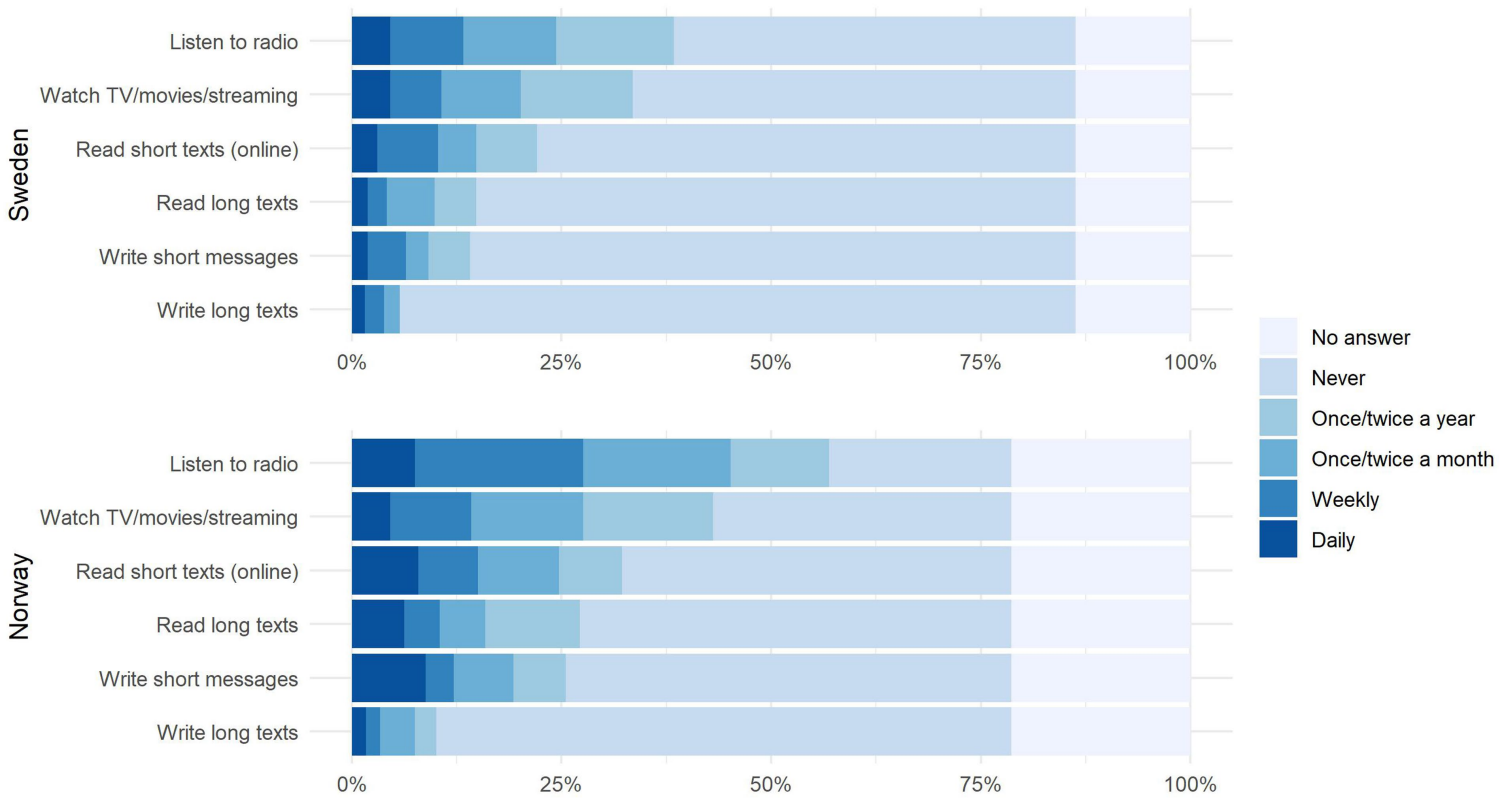
Frequency of Sámi language engagement outside the family (e.g., media and internet) for Norway (top) and Sweden (bottom).

To examine use *outside* the family, we asked for frequency of Sámi use in situations that are typically associated with higher levels of literacy (Figure 7). Receptive use (radio, TV, streaming) is high in both Norway and Sweden, with 57% of respondents in Norway listening to the radio in Sámi at least once or twice a year, and 39% in Sweden. Unsurprisingly, the least frequent activity is writing long texts (10% of respondents in Norway and 5% in Sweden). As with language use within the family, we performed statistical comparisons between Sweden and Norway for each activity separately. All comparisons were highly significant (*p* < .01; see Supplementary materials), again showing higher levels of use in Norway than in Sweden. The implications are discussed in section 5.

### 4.3. Results from North Sámi Vocabulary Task

In addition to the self-reported measures used in the survey, a small subset of participants who specifically indicated that they spoke North Sámi completed a vocabulary task in North Sámi (*n* = 44 in Sweden, and *n* = 38 in Norway). Since no easy-to-implement measure of North Sámi proficiency was available, we developed the North Sámi Vocabulary Task (NSVT) specifically for this purpose (see Gyllstad et al., in preparation, see footnote 3). The test was modelled on the DIALANG placement test (Alderson, 2005), and contained 50 real words and 25 pseudowords (see Supplementary materials).^10^ The items appeared on the screen one at a time, and participants were instructed to click ‘Yes’ if they thought the item existed, or ‘No’ if they did not. One point for each correctly-identified item was given, rendering a maximum score of 75.

Table 6 shows that the mean score on the NSVT is higher in Norway (55.97) than in Sweden (47.29). A simple linear regression indicated that the effect of ‘country’ on ‘vocabulary score’ is significant (β = −8.67, SE = 2.659, t = −3.263, *p* = .001). Assuming vocabulary knowledge can be taken as a proxy for general proficiency (e.g., Alderson, 2005), we can carefully conclude that North Sámi proficiency was higher in Norway. This might reflect the better educational policies in Norway, whereby children have better opportunities to learn the (written^11^) language at school.

**Table 6:** Scores on Sámi vocabulary task (NSVT) in Norway and Sweden.

	Mean	SD	Min	Max
North Sámi speakers in Sweden (*n* = 44)	47.29	12.77	25	72
North Sámi speakers in Norway (*n* = 38)	55.97	11.05	35	72

**Figure 8:**
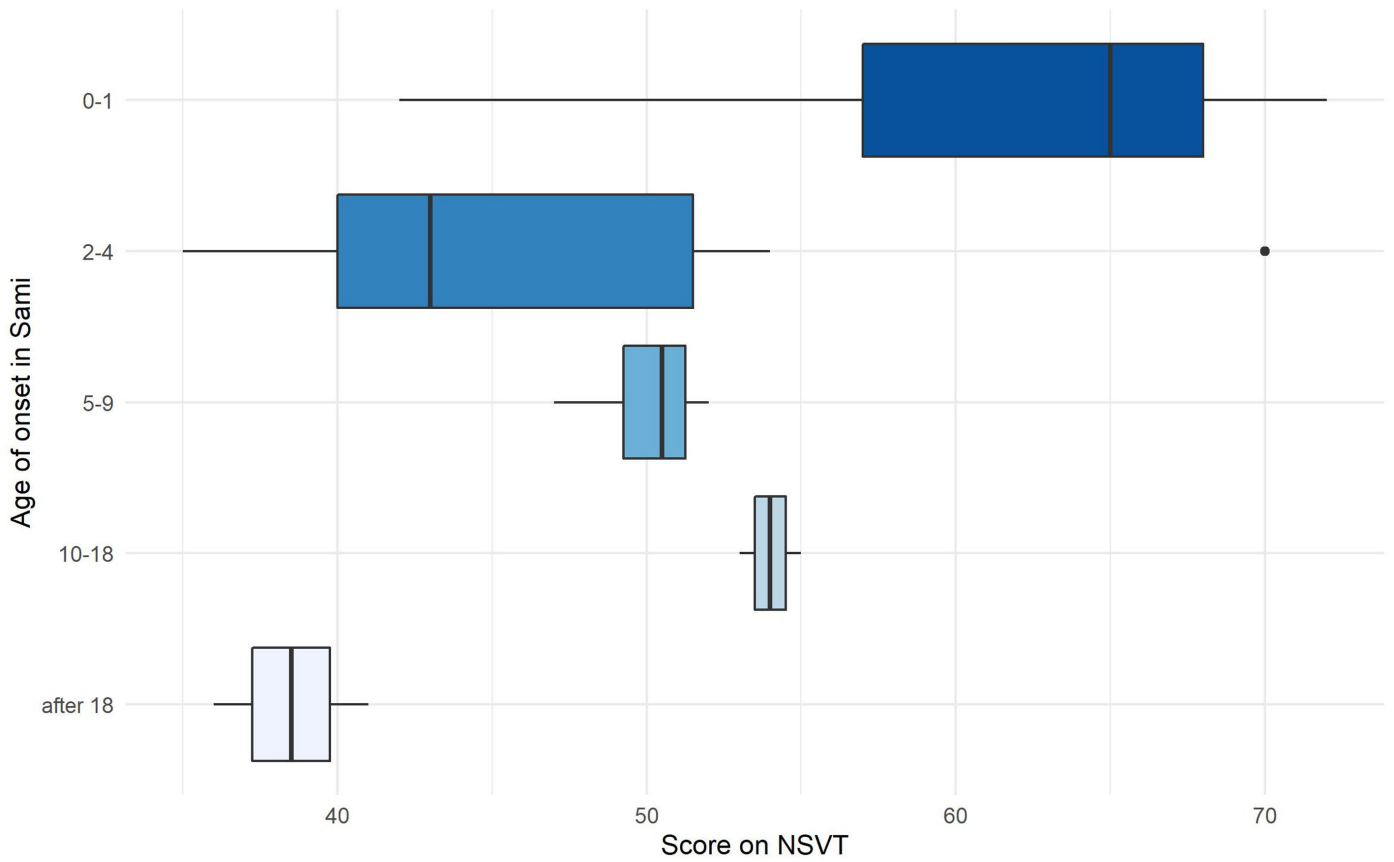
Result on NSVT for North Sámi participants (both countries) by age on onset in Sámi.

In an attempt to better understand which aspects of language experience were predictive of the vocabulary test scores, we looked at the effect of ‘age of first exposure to Sámi.’ We found that participants with a very early age of onset (i.e., between 0 and 1 years) achieved substantially higher scores on the test than participants who began acquiring Sámi as older children or adults. A basic linear regression showed that the difference between the ‘0-1 age group’ and all other age groups was significant (*p* < .01 or lower). This likely reflects the higher amount of input received by the ‘0-1 group’ as compared to the other groups (Figure 8).

## 5. Discussion

In the following, we interpret the main findings from the survey in light of our research questions and previous studies (sections 5.1–5.3). We end with a discussion on the role of policies (sections 5.4–5.5).

### 5.1. Language use across the generations

RQ1 was concerned with changes in Sámi language use across generations. In the data from the Stage I CATI interviews, we observed a drastic drop in self-reported Sámi use between the generation of the grandparents (Norway = 75% Sámi users, Sweden = 53% Sámi users), the parents, and the participants themselves (23% vs. 20% Sámi users). Here we see the systematic effects of the older generation, i.e., the grandparents of today, having been forced to give up their language at school, and not passing their language on to their children, the parents of today (Olthuis et al., 2013). However, more-detailed results from the Stage II survey give us reason to believe that there is a pushback against this trend in Sámi-speaking parents today. Namely, the rate of participants who reported having used ‘mostly Sámi’ with their parents as preschool children is approximately the same as the proportion of participants who today use ‘mostly Sámi’ with their *own* pre-school children (around 5% of Sámi respondents in Sweden and 10% in Norway). This suggests that those who received high amounts of linguistic input as children themselves, i.e., today’s fluent Sámi-speaking parents, generally pass their knowledge of Sámi on to the next generation. One possible explanation is that attitudes towards the Sámi languages have improved in today’s parents as compared to previous generations, the majority of whom did not pass the language on to their children.

### 5.2. Self-assessed Sámi proficiency in non-Sámi respondents

RQ2 addressed the levels of self-assessed language proficiency in non-Sámi respondents, who have not yet been the subject of investigation on Sámi matters to date. We showed that only 24% of Swedish non-Sámi and 36% of Norwegian non-Sámi participants reported being able to understand at least a ‘few words’ of a Sámi language. Considering that our respondents live in Sámi municipalities or in areas with a large share of Sámi residents, we think these numbers show that more work is needed to increase proficiency of Sámi in the majority population. Though self-assessed proficiency has to be taken with a grain of salt, we think these data are interesting, and they might reflect a higher aware of non-Sámi Norwegians in Sámi matters. Support for this idea can be found in (anecdotal) data we obtained in open comments fields, which we will report on briefly. We received a total of 19 comments from Swedish non-Sámi respondents, who indicated the question of Sámi at school was not relevant to them because they were not Sámi. However, we did not receive such comments from the non-Sámi Norwegian participants. This might suggest that Sámi learning is more widely seen in Norway as being relevant to everyone. For Sweden, this could be achieved by raising awareness of the Sámi culture in school education. Hettema and Outakoski (2020) points out that textbooks in Sweden do not contain ample information on national minorities, and also argues that more awareness building is needed in this area.

### 5.3. Language use and proficiency in Sámi respondents

RQ3 targeted language use and proficiency amongst the ethnic Sámi respondents. For self-assessed proficiency, just over half in Sweden (57%) and just over two thirds in Norway (70%) said they could understand at least ‘a few words’ of Sámi. This result lines up with the most positive estimates in the field, e.g., those given by the Ethnologue, who estimate that 25,000 of 30,000 Ethnic Sámi people are language users (Eberhard et al., 2022). However, our results suggest these estimates are not ‘users,’ but more accurately people with loose contacts to the language, who understand a few words. In contrast, less than one third (27%) of Sámi in Norway and less than one fifth (18%) in Sweden assessed their comprehension as *moderate* or better, i.e., as potentially good enough to follow a conversation. This finding is closer to estimates by Rasmussen and Nolan (2011) and Hettema and Outakoski (2020), who gauge that only around one third of the Sámi population are Sámi users. Native-like proficiency was specified by only 11.3% of participants in Norway, and 4.18% in Sweden.

We also asked about language use in various contexts, which has not yet been done in previous studies. Based on a battery of questions about language use with family members across the lifespan, we showed that in both countries, around 20% of the ethnic Sámi population uses Sámi at least *occasionally*. However, the proportion who used ‘mostly’ or ‘only’ Sámi was extremely low, at 10% in Norway and at less than 5% in Sweden. We believe that this is the proportion of ethnic Sámi who can be considered as fluent speakers of a Sámi language.

Further, we noted a surprisingly high amount of *receptive* Sámi use in both countries. Over a third of Swedish Sámi and over half of Norwegian Sámi listened to the radio, watched TV, or used streaming services at least occasionally. This finding highlights the important role that is played by such channels in providing sources of language input. At the same time, we also know that passive exposure in itself is insufficient, and needs to be complemented by active use. This is because receptive bilinguals are less likely to be able to pass their language on to the next generation.

We further think that the higher rates of receptive engagement with the Sámi languages in Norway is due to the better support that is given for cultural measures, as evidenced by the higher budget for the Norwegian Sámi Parliament. By its own account, *Samefolket* is the only Sámi newspaper in Sweden, and publishes the majority of its texts in Swedish. In contrast, there are several Sámi-language newspapers in Norway, such as *SÁMi magasiidna*^12^ and *Ávvir*. They receive considerable funding from the government. In fact, in 2013 *Ávvir* received the highest government subsidies of all newspapers in northern Norway.

To measure language proficiency directly and to test the reliability of our indirect, self-reported measures, we implemented a vocabulary task (NSVT) with a subset of participants who spoke North Sámi. Two findings are of interest: First, high test scores were generally only achieved by early bilinguals, who had acquired Sámi from birth. This was true for Sámi speakers in both countries, and reflects what we know from bilingualism research, namely that an early age of onset is crucial (Johnson and Newport, 1989; Abrahamsson and Hyltenstam, 2009), of course paired with high quality input (Chondrogianni and Marinis, 2011). Given the observation that only 10% of ethnic Sámi participants in Norway and less than 5% in Sweden use Sámi regularly with their children, we can conclude that current levels of language transmission are likely to be insufficient for the language to survive. In both countries, there is a need for a larger number of L2 learners—both Sámi and non-Sámi—and the need to work on raising the perceived importance of the Sámi languages in majority society. Second, we once again found significantly higher scores in Norway than in Sweden. Of course, this might be a reflection of the larger North-Sámi community in Norway than in Sweden, and it is likely that we would receive better results in Sweden for a test of South or Lule Sámi.

### 5.4. Policies as a motor for language experience

With RQ4 we examined whether potential differences between the two countries could be explained by their respective policies. Obviously, one-shot observational data like ours do not allow for a verifiable analysis of policy effects. Instead, the aim of this paper is to present novel data on Sámi language use and proficiency, and to elaborate on how these might be associated with themes found in the national Sámi policies.

Indeed, it seems the higher language use and proficiency levels we found in Norway may reflect educational policies. In Norway, there is a larger number of schools that offer Sámi lessons for a longer period of time and our data confirm that there is also a larger proportion of Sámi who learn a Sámi language at school (a total of 22.17% of Norwegian Sámi reported having had some form of Sámi lessons at school, but only 13.98% of Swedish Sámi respondents did so). As a result, Sámi-medium education should be extended to the high school years in Sweden (Belančić and Lindgren, 2020), as is already the case in Norway. At the same time, even in Norway existing policies may be insufficient. Vangsnes (2022) recently pointed out that, even if all school learners of Sámi became ‘bearers’ of the language and carry the language forward in time, the total number of Sámi speakers is likely to drop unless additional measures are taken, such as increasing the number of L2 learners, and making improvements to the curriculum. Further, as suggested by Albury (2015), Sámi learning at school needs to be made relevant to *all* citizens, including non-Sámi. Finally, it is conceivable that immersive teaching (i.e., a Sámi track in which all teaching is in Sámi) would be needed to create new speakers, as done for example in the Māori language in New Zealand (Hill and May, 2014).

Our analysis of budgetary spending showed that the Sámi parliament in Norway allocates a larger proportion of their budget to language and culture than in Sweden. Budgetary policies have a both symbolic and tangible effect on language experience. Although the total budget of the Sámi Parliament in Sweden is increasingly approaching that of Norway, the share of the budget that is related to the promotion of Sami languages seems to be declining slightly. The amount of financing available for the promotion of the Sámi languages conveys a message about how much the languages are valued, and spending a higher proportion of public resources on the Sámi languages signals a higher societal appreciation of the languages. More practically, a higher budget means more funding for producing radio and TV-shows, publishing books and newspapers in Sámi, and for other language undertakings. Taken together, the differences we found between the two countries in data seem to reflect the two countries’ Sámi language policies. Norway’s national Sámi language policies provide more comprehensive Sámi education opportunities and more monetary resources for Sámi language work, thus conveying a more affirmative status message about the languages to the overall public. As a result, we also see more frequent use of Sámi languages and higher proficiency levels (self-assessed and objective) in Norway.

It also seems likely that Norway’s move to make Sámi the co-official language has positively impacted on the awareness and acceptance of the Sámi languages amongst the non-Sámi population, who were shown in our study to be more likely to learn a Sámi language at school, and to have a higher level of Sámi language proficiency. Although some measures have been undertaken in Sweden, it seems paramount that awareness of the Sámi languages is increased in the majority population. One strategy could be higher budgetary allocations to language and a higher legal status of the Sámi languages. As mentioned earlier on, the EGIDS places the North Sámi language in Sweden on Stage 6, meaning that intergenerational transmission is taking part, but is not necessarily secure. Therefore, such measures need to be taken in parallel to strengthening Sámi language use in families. Otherwise, top-down measures introduced in isolation could threaten to institutionalise the language, rather than elevating the position of the Sámi languages to languages that are acquired as L1s by young children.

### 5.5. Limitations

Admittedly, there are several limitations to these conclusions. Based on our analysis it is not possible to make a statement about the causal nature of these effects. The significant differences we found between Norway and Sweden for almost all measures might be due to the larger speaker community in the former country. At the same time, it may be that the larger speaker community is there due to the political support. Further, regardless of how good a policy may be, its ultimate success on the ground depends on complex societal conditions. For example, Sámi revitalization policies are challenged by increased globalisation and speaker mobility, whereby people who would benefit from these policies no longer live in the traditional Sámi areas, and in which multicultural family constellations make language transmission increasingly difficult. A further point is that, even if policies towards a minority group are improved, the negative attitudes towards these languages (and, by implication, towards the people who speak them) may be entrenched. Such persisting attitudes may prevent people from using and developing their minority language. Therefore, policy measures must go hand in hand with measures to increase the visibility and appreciation, also in non-Sámi areas. Ideally, Sámi and non-Sámi school students should be proud of learning a Sámi language, and they should be rewarded for doing so. Learning Sámi should become as attractive as learning any (foreign) language. Finally, it is worth pointing out that our study was carried out in the Northern provinces where there is generally more awareness of Sámi matters. For future studies, it would be interesting to look at awareness in the Southern provinces, where we would expect even more drastic differences.

## 6. Conclusion

This paper investigated language maintenance in two countries that aim at promoting and protecting their Indigenous languages. Our study combined a policy analysis with questionnaire data and proficiency measures, allowing a triangulation of findings across methods. The policy analysis shows that while both countries’ official aims are essentially the same, i.e., to promote and protect their minority languages, Norway seems to be committed somewhat more strongly, evidenced by its stronger educational policies, its budgetary spending, and the status of Sámi as an official language. The policy context in Norway leads to a better visibility of the Sámi languages in Norwegian society. The language use and proficiency data show that less than a third of Norwegian Sámi and a fifth of Swedish Sámi seem to know their Sami language well enough to follow a conversation in this language, and only 10% in Norway and less than 5% in Sweden can use the language fluently. Our data show that there are clearly more opportunities for engagement with the Sámi languages in Norway than in Sweden, and a higher proportion of speakers who are fluent enough to be able to pass the language on to their own children. We suggest that both countries need to increase their policy efforts by providing more opportunities for Sámi language education, increasing the proportion of financial resources for language and cultural spending, and generally signalling a higher appreciation for the Sámi languages in the majority society. Despite the many hurdles that still need to be overcome, it seems that commitment to Indigenous language revitalisation pays off.

## Data availability statement

The raw data supporting the conclusions of this article will be made available by the authors, without undue reservation.

## Ethics statement

The data collection for this study was reviewed and approved by the University of Konstanz Ethics Committee and the Swedish *Etikprövningsmyndigheten* [Ethical Review Authority] (project reference number: 2020-06853).

## Author contributions

AL-S and TK contributed to conception and design of the study. AL-S and FB performed the statistical analysis and wrote the first draft of the manuscript. LH, FB, and TK wrote sections of the manuscript. All authors contributed to the article and approved the submitted version.

## Funding

Data collection for this study was funded by the *Deutsche Forschungsgemeinschaft* (DFG—German Research Foundation) under Germany’s Excellence Strategy—EXC-2035/1—390681379.

## Conflict of interest

The authors declare that the research was conducted in the absence of any commercial or financial relationships that could be construed as a potential conflict of interest.

## Publisher’s note

All claims expressed in this article are solely those of the authors and do not necessarily represent those of their affiliated organizations, or those of the publisher, the editors and the reviewers. Any product that may be evaluated in this article, or claim that may be made by its manufacturer, is not guaranteed or endorsed by the publisher.

## Footnotes

^1^In a future study, examining Finland would be interesting, too, especially given that Inari Sámi, though spoken by fewer individuals, is viewed as reasonable stable (Eberhard et al., 2022), and also because Sámi is typologically close to Finnish. See Olthuis et al. (2013) for an account of the revitalization of Inari Sámi in Finland.

^2^Within this field, many tools have been developed that can help researchers to quantify the amount of language use early bilinguals have had across their lifespans (e.g., the UBiLEC, for use with child-aged early bilinguals, Unsworth, 2013; the Leap-Q, Marian et al., 2007; the Bilingual Language Profile; Gertken et al., 2014; the LSBQ, Anderson et al., 2018). Our questionnaire was inspired by Lloyd-Smith et al. (2017) and Kupisch et al. (2020) because it was specifically geared towards assessing language experience in adult minority language speakers.

^3^Gyllstad, H., Kupisch, T., and Lloyd-Smith, A. (in preparation). NorSamVoc — A lexical assessment tool for North Sámi.

^4^The denomination for the Norwegian area is *forvaltningsområdet for samisk språk*. Since 2020 it encompasses the municipalities of Karasjok, Kautokeino, Nesseby, Porsanger, Tana, Kåfjord, Lavangen, Tjeldsund, Hattfjelldal, Hamarøy, Røyrvik, Røros and Snåsa (Forskrift om endring i forskrift 17. juni, 2005 nr. 657 til sameloven (lov 12. juni 1987 nr. 56) om forvaltningsområdet for samisk språk, 2020). In Sweden, the *förvaltningsområdet för samiska* since 2019 comprises 25 municipalities, namely Arjeplog, Arvidsjaur, Berg, Dorotea, Gällivare, Härjedalen, Jokkmokk, Kiruna, Krokom, Luleå, Lycksele, Malå, Sorsele, Stockholm, Storuman, Strömsund, Sundsvall, Umeå, Vilhelmina, Vindeln, Åre, Åsele, Älvdalen, Örnsköldsvik and Östersund (Sametinget, 2019).

^5^Yasar, R., Lloyd-Smith, A., Bergmann, F., Schmid, S.-P., Kupisch, T., and Holzinger, K. (in preperation). Experience of Discrimination in Egalitarian Societies: The Sámi and Majority Populations in Sweden and Norway. Ethnic and Racial Studies.

^6^While both countries adopted the UNDRIP from the outset in 2007, Norway was earlier in signing and ratifying the ECRML (in 1992/1993 vs. 2000 in Sweden).

^7^The differences in response rates were likely due to the different modus operandi of the local survey companies. Alternatively, survey fatigue might have been higher in the Norwegian municipalities, since other Sámi-issue surveys, such as those of the SAMINOR project (*cf.* Lund et al., 2007; Brustad et al., 2014), have been previously conducted in these municipalities.

^8^We are aware that survey research comes with a risk of flawed response behaviour. However, by connecting the two stages, this risk was reduced. Respondents who were less committed to participating had the option to drop out after the CATI. We assume that those who continued with Stage II were interested in the study, and were likely to answer earnestly.

^9^The questions were open to speakers of all Sámi languages and for the purposes of this paper, we do not differentiate between speakers of, e.g., North vs. South Sámi (but see Supplementary materials for the data on which Sámi languages were spoken). North Sámi was the main language spoken by 94.2% of respondents in Norway and 63.2% in Sweden. These numbers match the most recent estimates from the Sámi Parliament (Sønstebø, 2021). In Sweden, we further found speakers of South Sámi in Storuman and Västerbotten, and Lule Sámi in Norrbotten. Surprisingly, several speakers of Pite and Ume Sámi (both moribund languages) were found in Arjeplog, Storuman and Sorsele. This matches the information provided by the Ethnologue (Eberhard et al., 2022), which places a small handful of Pite and Ume speakers precisely in these areas.

^10^The real-word items were obtained by translating and piloting the verbs from the Finnish, Norwegian and Swedish DIALANG placement tests into North Sámi; the pseudowords were constructed and piloted with the help of a highly proficient Sámi speaker, who was a linguist. Non-Sámi participants were excluded from analyses, since their scores on the test were in most cases below chance level (37.5/75 or less).

^11^Although reading involved only individual words, we are aware that one limitation of this proficiency measure is that it is arguably less suitable for speakers with lower literacy levels. At the same time, it is a method than can easily be used online.

^12^*SÀMi magasiidna* had to file for bankruptcy in July 2022. However, when our survey was conducted (April–September 2021) it was still an active media outlet.
