# Supplementary material for: Can policies improve language vitality? The Sámi languages in Sweden and Norway
Source: Front Psychol. 2023 Mar 29;14:1059696. doi: 10.3389/fpsyg.2023.1059696 (PMC10187035; doi:10.3389/fpsyg.2023.1059696)
Supplement: Supplementary file 1 [file Data_Sheet_1.pdf]

Supplementary material for:

Lloyd-Smith, A., Bergmann, F., Hund, L., & Kupisch, T. (2023). Can policies improve language vitality? The Sámi languages in Sweden and Norway. *Frontiers in Psychology. Section Language Sciences.* Vol. 14.

APPENDIX A

The following tables list the Norwegian and Swedish municipalities where the survey was conducted and their adult population figures. This information comes from the Norwegian and Swedish national statistical offices. The tables further list the absolute and relative numbers of survey respondents in each municipality and the number of speakers of the Sámi varieties among them. Municipalities in italics are part of the Sámi administrative areas in their respective country.

Table A1. Population, respondents, and speaker numbers by Sámi language and municipality – Norway

Municipality	Adult population as of 2017	Total respondents	Proportion	North Sámi	South Sámi	Lule Sámi
Alta	15458	295	1.91%	26		
Deatnu – Tana	2396	26	1.09%	13		
Divtasvuodna – Tysfjord	1623	18	1.11%	2		1
Guovdageaidnu – Kautokeino	2290	15	0.66%	10	1	
Gáivuotna – Kåfjord – Kaivuono	1774	12	0.68%	4		
Kvalsund	862	4	0.46%			3
Kvænangen	1014	17	1.68%			
Kárásjohka – Karasjok	2175	12	0.55%	11		
Lebesby	1076	17	1.58%	2		
Loábak – Lavangen	829	7	0.84%	2		
Lyngen	2342	14	0.60%	0		
Porsanger – Porsángu – Porsanki	3245	42	1.29%	9		
Raarvihke – Røyrvik	382	1	0.26%	0		
Storfjord – Omasvuotna	1523	16	1.05%	0		
Unjárga – Nesseby	778	6	0.77%	4		
Total	37767	502	1.33%	83	1	4

Table A2. Population, respondents, and speaker numbers by Sámi language and municipality – Sweden

Municpality	Adult population as of 2017	Total respondents	Proportion	North Sámi	South Sámi	Lule Sámi	Ume Sámi	Pite Sámi
*Arjeplog**	2364	64	2.71%	5	1	2	2	5
Jokkmokk	4257	86	2.02%	9	1	8		
Kiruna	18637	503	2.70%	92	1	9		1
Sorsele	2068	35	1.69%				5	
Storuman	4879	133	2.73%	4	19		9	
Total	32205	821	2.55%	110	22	19	16	6

APPENDIX B

The following tables list the observed distribution of data and corresponding Pearson's chi-squared tests of independence related to Figures 3 to 7 of the article. The test statistics indicate whether the distributions differ between Norway and Sweden on a statistically significant level.

Table B1.1. Self-assessed proficiency *(Figure 3)* – Non-Sámi respondents

	**Norway**	**Sweden**	∑
Nativelike	2	1	*3*
Very well	3	3	*6*
Moderately	7	11	*18*
A few words	84	143	*227*
Not at all	137	421	*558*
∑	*233*	*579*	*812*
$\chi2$ = 16.7006** (df = 4)			

* p < 0.05, ** p < 0.01, *** p < 0.001

Table B1.2. Self-assessed proficiency *(Figure 3)* – Sámi respondents

	**Norway**	**Sweden**	∑
Nativelike	27	11	*38*
Very well	7	7	*14*
Moderately	29	28	*57*
A few words	101	104	*205*
Not at all	42	77	*119*
∑	*206*	*227*	*433*
χ^2 = 16.1118** (df = 4)			

* p < 0.05, ** p < 0.01, *** p < 0.001

Table B2.1. Self-reported Sámi learning at school *(Figure 4)* – Non-Sámi respondents

	**Norway**	**Sweden**	∑
We had some foreign language classes	7	5	*12*
The instruction of some subjects was in Sámi	1	0	*1*
No Sámi at school	209	486	*695*
Other/skipped question	16	101	*117*
∑	*233*	*592*	*825*
$\chi^2 = 21.3010^{***}$ (df = 2)			

* p < 0.05, ** p < 0.01, *** p < 0.001

Note: Due to the very small number of non-Sámi respondents stating they learned Sámi at school this, Chi-squared test has little informative value, even though it is highly significant. Answer patterns differ between Norway and Sweden primarily in the category “Other/skipped question”.

Table B2.2. Self-reported Sámi learning at school *(Figure 4)* – Sámi respondents

	**Norway**	**Sweden**	∑
We had some foreign language classes	33	29	*62*
The instruction of some subjects was in Sámi	14	4	*18*
No Sámi at school	147	155	*302*
Other/skipped question	18	48	*66*
∑	*212*	*236*	*448*
$\chi^2 = 18.4291^{***}$ (df = 2)			

* p < 0.05, ** p < 0.01, *** p < 0.001

Table B3.1. Amount of Sámi spoken before age 6 *(Figures 5 and 6)* – With mother

	**Norway**	**Sweden**	∑
Only Sámi	22	9	*31*
Mostly Sámi	1	3	*4*
Half-half	4	3	*7*
Mostly majority	6	29	*35*
Never	165	187	*352*
∑	*198*	*231*	*429*
$\chi^2 = 31.8745^{***}$ (df = 4)			

* p < 0.05, ** p < 0.01, *** p < 0.001

Table B3.2. Amount of Sámi spoken before age 6 *(Figures 5 and 6)* – With father

	**Norway**	**Sweden**	∑
Only Sámi	25	10	*35*
Mostly Sámi	0	2	*2*
Half-half	2	2	*4*
Mostly majority	6	32	*38*
Never	160	180	*340*
∑	*193*	*226*	*419*

$\chi^2 = 39.6531^{***}$ (df = 4)

* p < 0.05, ** p < 0.01, *** p < 0.001

Table B3.3. Amount of Sámi spoken before age 6 *(Figures 5 and 6)* – With grandparents

	**Norway**	**Sweden**	∑
Only Sámi	18	13	*31*
Mostly Sámi	2	5	*7*
Half-half	16	5	*21*
Mostly majority	17	38	*55*
Never	134	150	*284*
∑	*187*	*211*	*398*

$\chi^2 = 27.3426^{***}$ (df = 4)

* p < 0.05, ** p < 0.01, *** p < 0.001

Table B3.4. Amount of Sámi spoken before age 6 *(Figures 5 and 6)* – With siblings

	**Norway**	**Sweden**	∑
Only Sámi	21	8	*29*
Mostly Sámi	3	1	*4*
Half-half	2	3	*5*
Mostly majority	4	16	*20*
Never	166	194	*360*
∑	*196*	*222*	*418*

$\chi^2 = 21.2746^{***}$ (df = 4)

* p < 0.05, ** p < 0.01, *** p < 0.001

Table B3.5. Amount of Sámi spoken before age 6 *(Figures 5 and 6)* – With own children

	**Norway**	**Sweden**	∑
Only Sámi	12	6	*18*
Mostly Sámi	6	2	*8*
Half-half	3	2	*5*
Mostly majority	13	23	*36*
Never	133	152	*285*
∑	*167*	*185*	*352*
$\chi^2 = 7.2318$ (df = 4)			

* p < 0.05, ** p < 0.01, *** p < 0.001

Table B4.1. Amount of Sámi spoken currently *(Figures 5 and 6)* – With mother

	**Norway**	**Sweden**	∑
Only Sámi	14	6	*20*
Mostly Sámi	2	1	*3*
Half-half	5	1	*6*
Mostly majority	8	27	*35*
Never	138	165	*303*
∑	*167*	*200*	*367*
$\chi^2 = 21.1470$*** (df = 4)			

* p < 0.05, ** p < 0.01, *** p < 0.001

Table B4.2. Amount of Sámi spoken currently *(Figures 5 and 6)* – With father

	**Norway**	**Sweden**	∑
Only Sámi	15	5	*20*
Mostly Sámi	4	1	*5*
Half-half	6	3	*9*
Mostly majority	4	27	*31*
Never	131	156	*287*
∑	*160*	*192*	*352*
$\chi^2 = 25.5092$*** (df = 4)			

* p < 0.05, ** p < 0.01, *** p < 0.001

Table B4.3. Amount of Sámi spoken currently *(Figures 5 and 6)* – With grandparents

	**Norway**	**Sweden**	∑
Only Sámi	9	9	*18*
Mostly Sámi	2	0	*2*
Half-half	1	6	*7*
Mostly majority	20	7	*27*
Never	133	103	*236*
∑	*165*	*125*	*290*
$\chi^2 = 14.3990^{**}$ (df = 4)			

* p < 0.05, ** p < 0.01, *** p < 0.001

Table B4.4. Amount of Sámi spoken currently *(Figures 5 and 6)* – With siblings

	**Norway**	**Sweden**	∑
Only Sámi	16	7	*23*
Mostly Sámi	5	2	*7*
Half-half	4	1	*5*
Mostly majority	8	21	*29*
Never	159	190	*349*
∑	*192*	*221*	*413*
$\chi^2 = 15.2674^{**}$ (df = 4)			

* p < 0.05, ** p < 0.01, *** p < 0.001

Table B4.5. Amount of Sámi spoken currently *(Figures 5 and 6)* – With own children

	**Norway**	**Sweden**	∑
Only Sámi	10	5	*15*
Mostly Sámi	6	2	*8*
Half-half	2	3	*5*
Mostly majority	11	28	*39*
Never	136	147	*283*
∑	*165*	*185*	*350*
$\chi^2 = 10.0118^{*}$ (df = 4)			

* p < 0.05, ** p < 0.01, *** p < 0.001

Table B5.1. Frequency of Sámi language engagement outside the family *(Figure 7)* – Listen to radio

	**Norway**	**Sweden**	∑
Daily	18	12	*30*
Weekly	48	23	*71*
Once/twice a month	42	29	*71*
Once/twice a year	28	37	*65*
Never	52	126	*178*
No answer	51	36	*87*
∑	*239*	*263*	*502*

$\chi^2 = 45.9371^{***}$ (df = 5)

* p < 0.05, ** p < 0.01, *** p < 0.001

Table B5.2. Frequency of Sámi language engagement outside the family *(Figure 7)* – Watch TV

	**Norway**	**Sweden**	∑
Daily	11	12	*23*
Weekly	23	16	*39*
Once/twice a month	32	25	*57*
Once/twice a year	37	35	*72*
Never	85	139	*224*
No answer	51	36	*87*
∑	*239*	*263*	*502*

$\chi^2 = 16.7099^{**}$ (df = 5)

* p < 0.05, ** p < 0.01, *** p < 0.001

Table B5.3. Frequency of Sámi language engagement outside the family *(Figure 7)* – Read short texts

	**Norway**	**Sweden**	∑
Daily	19	8	*27*
Weekly	17	19	*36*
Once/twice a month	23	12	*35*
Once/twice a year	18	19	*37*
Never	111	169	*280*
No answer	51	36	*87*
∑	*239*	*263*	*502*

$\chi^2 = 21.5792^{***}$ (df = 5)

* p < 0.05, ** p < 0.01, *** p < 0.001

Table B5.4. Frequency of Sámi language engagement outside the family *(Figure 7)* – Read long texts

	**Norway**	**Sweden**	∑
Daily	15	5	*20*
Weekly	10	6	*16*
Once/twice a month	13	15	*28*
Once/twice a year	27	13	*40*
Never	123	188	*311*
No answer	51	36	*87*
∑	*239*	*263*	*502*

$\chi^2 = 26.1266^{***}$ (df = 5)

* p < 0.05, ** p < 0.01, *** p < 0.001

Table B5.5. Frequency of Sámi language engagement outside the family *(Figure 7)* – Write short messages

	**Norway**	**Sweden**	∑
Daily	21	5	*26*
Weekly	8	12	*20*
Once/twice a month	17	7	*24*
Once/twice a year	15	13	*28*
Never	127	190	*317*
No answer	51	36	*87*
∑	*239*	*263*	*502*
$\chi^2 = 28.9812^{***}$ (df = 5)			

* p < 0.05, ** p < 0.01, *** p < 0.001

Table B5.6. Frequency of Sámi language engagement outside the family *(Figure 7)* – Write long texts

Write long texts (letters, essays)	**Norway**	**Sweden**	∑
Daily	4	4	*8*
Weekly	4	6	*10*
Once/twice a month	10	5	*15*
Once/twice a year	6	0	*6*
Never	164	212	*376*
No answer	51	36	*87*
∑	*239*	*263*	*502*
$\chi^2 = 15.6690^{**}$ (df = 5)			

* p < 0.05, ** p < 0.01, *** p < 0.001

APPENDIX C

Table C1. Items and item statistics for the North Sámi vocabulary task (NSVT)

Item no.	Item	Criteria	r² (binomial)	Facility (%)	DiscrimValue	Frequency*
SA01	beahkat	pseudoword	0.4379416	71,43	1,00	n.a.
SA02	áhppet	pseudoword	0.4238612	64,29	0,67	n.a.
SA03	noađđit	pseudoword	0.2654192	71,43	0,33	n.a.
SA04	arddašit	pseudoword	0.3693048	64,29	0,67	n.a.
SA05	jienastuvvat	pseudoword	0.165887	35,71	0,33	n.a.
SA06	čalmmuhit	pseudoword	0.03908329	14,29	0,33	n.a.
SA07	láhppat	pseudoword	0.7679075	57,14	1,00	n.a.
SA08	láidut	pseudoword	0.3944847	42,86	0,33	n.a.
SA09	lohkahallat	pseudoword	0.5631322	50,00	0,67	n.a.
SA10	váivašuhttit	pseudoword	0.4748952	28,57	0,33	n.a.
SA11	buoskudit	pseudoword	0.2874702	64,29	0,33	n.a.
SA12	jápmidit	pseudoword	0.4218372	57,14	0,33	n.a.
SA13	gáskut	pseudoword	0.7218231	42,86	0,67	n.a.
SA14	juohkašit	pseudoword	0.589753	50,00	1,00	n.a.
SA15	roššut	pseudoword	0.7829866	71,43	1,00	n.a.
SA16	johkat	pseudoword	0.456649	50,00	1,00	n.a.
SA17	čuollit	pseudoword	0.722857	50,00	1,00	n.a.
SA18	olmmuhit	pseudoword	0.7679075	57,14	1,00	n.a.
SA19	skáiput	pseudoword	*NA*	100,00	0,00	n.a.
SA20	geahnodit	pseudoword	0.3755549	78,57	0,67	n.a.
SA21	veajit	pseudoword	0.4372382	64,29	0,67	n.a.
SA22	árbalit	pseudoword	0.2366654	71,43	0,67	n.a.
SA23	iloštuvvat	pseudoword	0.5745955	42,86	0,67	n.a.
SA24	ávžidit	pseudoword	0.3755549	78,57	0,33	n.a.
SA25	biednut	pseudoword	0.5645437	78,57	0,67	n.a.
SA26	addit	real word	0.5429618	92,86	0,33	14584
SA27	nannet	real word	0.6875543	78,57	1,00	618
SA28	šaddat	real word	0.5429618	92,86	0,33	12216
SA29	mearridit	real word	0.4096028	92,86	0,33	9840
SA30	ohcat	real word	0.7010685	85,71	0,33	8964
SA31	bivdit	real word	*NA*	100,00	0,00	6585
SA32	hukset	real word	0.6029189	85,71	0,33	5498
SA33	lasihit	real word	0.8549588	78,57	0,67	4652
SA34	dieđihit	real word	*NA*	100,00	0,00	4700
SA35	almmuhit	real word	*NA*	100,00	0,00	4044
SA36	deaivvadit	real word	*NA*	100,00	0,00	3562
SA37	orrut	real word	00.04481	92,86	0,33	4112
SA38	juohkit	real word	*NA*	100,00	0,00	3368
SA39	jearrat	real word	0.5429618	92,86	0,33	3368
SA40	vuoitit	real word	0.5829264	78,57	0,33	3185
SA41	áddet	real word	0.1428847	92,86	0,33	2878
SA42	jienastit	real word	0.5429618	92,86	0,33	2448
SA43	gokčat	real word	0.6029189	85,71	0,33	1791
SA44	viiddidit	real word	0.7782219	64,29	1,00	1777
SA45	čatnat	real word	0.4066198	85,71	0,33	1419
SA46	cealkit	real word	0.3084702	85,71	0,33	1308

SA47	ovttastahttit	real word	0.4155219	78,57	0,67	1093
SA48	gieldit	real word	0.852947	78,57	0,67	1019
SA49	mieđihit	real word	0.7423827	64,29	0,67	997
SA50	doapmat	real word	0.7010685	85,71	0,67	243
SA51	gierdat	real word	0.2103206	85,71	0,33	632
SA52	boktit	real word	0.1428847	92,86	0,33	591
SA53	báhtarit	real word	0.2416975	85,71	0,33	618
SA54	bálkestit	real word	*NA*	100,00	0,00	622
SA55	coggat	real word	*NA*	100,00	0,00	542
SA56	johtalit	real word	0.1737745	71,43	0,67	358
SA57	oađđit	real word	*NA*	100,00	0,00	459
SA58	čiehkat	real word	*NA*	100,00	0,00	346
SA59	gaikut	real word	0.6029189	85,71	0,33	297
SA60	geardduhit	real word	0.5429618	85,71	0,33	516
SA61	dovddastit	real word	0.5410346	92,86	0,33	880
SA62	ribahit	real word	0.1428847	92,86	0,33	226
SA63	unnut	real word	0.8532938	71,43	0,67	152
SA64	botnjat	real word	0.5158931	71,43	0,67	143
SA65	duolbmat	real word	0.5829264	78,57	0,33	140
SA66	gollat	real word	0.5201497	78,57	0,33	135
SA67	bordit	real word	0.8532938	71,43	0,67	115
SA68	háleštit	real word	0.4066198	85,71	0,33	1225
SA69	nágget	real word	0.07874158	71,43	0,33	77
SA70	ráfehuhttit	real word	0.4464877	50,00	0,33	46
SA71	suođđat	real word	0.1510365	64,29	0,33	37
SA72	gáskit	real word	*NA*	100,00	0,00	33
SA73	garret	real word	0.5452673	64,29	0,33	31
SA74	jiekŋut	real word	0.7249656	71,43	0,67	29
SA75	ruohttat	real word	0.7010685	85,71	0,67	163

Note: *Frequency = absolute values from the SIKOR corpus
